# An E2F1-Mediated DNA Damage Response Contributes to the Replication of Human Cytomegalovirus

**DOI:** 10.1371/journal.ppat.1001342

**Published:** 2011-05-12

**Authors:** Xiaofei E, Mary T. Pickering, Michelle Debatis, Jonathan Castillo, Alexander Lagadinos, Shixia Wang, Shan Lu, Timothy F. Kowalik

**Affiliations:** 1 Department of Microbiology and Physiological Systems, University of Massachusetts Medical School, Worcester, Massachusetts, United States of America; 2 Program in Immunology and Virology, University of Massachusetts Medical School, Worcester, Massachusetts, United States of America; 3 Laboratory of Nucleic Acid Vaccines, Department of Medicine, University of Massachusetts Medical School, Worcester, Massachusetts, United States of America; Oregon Health and Science University, United States of America

## Abstract

DNA damage resulting from intrinsic or extrinsic sources activates DNA damage responses (DDRs) centered on protein kinase signaling cascades. The usual consequences of inducing DDRs include the activation of cell cycle checkpoints together with repair of the damaged DNA or induction of apoptosis. Many DNA viruses elicit host DDRs during infection and some viruses require the DDR for efficient replication. However, the mechanism by which DDRs are activated by viral infection is poorly understood. Human cytomegalovirus (HCMV) infection induces a DDR centered on the activation of ataxia telangiectasia mutated (ATM) protein kinase. Here we show that HCMV replication is compromised in cells with inactivated or depleted ATM and that ATM is essential for the host DDR early during infection. Likewise, a downstream target of ATM phosphorylation, H2AX, also contributes to viral replication. The ATM-dependent DDR is detected as discrete, nuclear γH2AX foci early in infection and can be activated by IE proteins. By 24 hpi, γH2AX is observed primarily in HCMV DNA replication compartments. We identified a role for the E2F1 transcription factor in mediating this DDR and viral replication. E2F1, but not E2F2 or E2F3, promotes the accumulation of γH2AX during HCMV infection or IE protein expression. Moreover, E2F1 expression, but not the expression of E2F2 or E2F3, is required for efficient HCMV replication. These results reveal a novel role for E2F1 in mediating an ATM-dependent DDR that contributes to viral replication. Given that E2F activity is often deregulated by infection with DNA viruses, these observations raise the possibility that an E2F1-mediated mechanism of DDR activation may be conserved among DNA viruses.

## Author Summary

As intracellular parasites, viruses often redirect cellular pathways to facilitate their own replication. Infection by DNA viruses often lead to the activation of host DNA damage response pathways, which normally function to repair damage to host chromosomes. Some DNA viruses depend on this infection-induced DNA damage response to efficiently replicate. How infection activates the DNA damage response is poorly understood. To address this limitation, we first determined whether the DNA damage response affects the replication of human cytomegalovirus (HCMV) and then addressed how infection induces this response in cells. We find that HCMV infection results in a host DNA damage response centered on the Ataxia Telangiectasia Mutated (ATM) protein kinase. We also show that HCMV requires ATM for efficient replication. Unexpectedly, we find that the mechanism responsible for ATM activation is the expression of E2F1, a cellular transcription factor. Moreover, expression of E2F1, like ATM, is required for HCMV replication. These observations may be of fundamental importance because infection by most DNA viruses result in both E2F1 expression and an ATM-mediated DNA damage response.

## Introduction

Cellular DNA is constantly bombarded by insults from both intrinsic sources, such as reactive oxygen species, and extrinsic sources, like genotoxic chemicals. DNA damage resulting from these challenge produces a complex protein kinase signaling cascade that promotes repair of the damaged DNA and activates cell cycle checkpoints or apoptosis [1]. A central mediator of certain DNA damage response (DDR) pathways is the ataxia telangiectasia mutated (ATM) protein kinase [2]. ATM activation leads to the phosphorylation of numerous proteins that ultimately signal cell cycle arrest and DNA repair and/or apoptosis. Recent data have shown that several viruses, including herpes simplex virus type 1 (HSV-1), polyomavirus, human papillomavirus (HPV), and human immunodeficiency virus type 1 (HIV-1) require the activation of ATM and/or downstream proteins for a fully permissive infection [3], [4], [5], [6], [7]. Presumably, these viruses also encode proteins that interfere with downstream DDR signaling that antagonize virus replication through the activation of cell cycle checkpoints or the induction of apoptosis.

Human cytomegalovirus (HCMV) infection activates multiple DDR proteins, including ATM and the downstream effector protein, p53 [8], [9], [10]. The p53 transcription factor plays an important role in responding to certain cellular stresses as well as in regulating cell cycle progression. It has been proposed that the activation of p53 helps to elicit the cell cycle arrest in HCMV infected fibroblasts by modulating p21 levels [11] or by facilitating viral gene expression [12]. However, the functional relevance of ATM in HCMV replication has been unclear. Although others have concluded that ATM does not contribute to HCMV replication [9], it seems reasonable to reconsider the role of ATM in this process given that downstream factors of ATM activation are required for efficient replication of HCMV and that ATM contributes to the replication of other DNA viruses.

It has been noted that the cellular environment of HCMV infected cells is “G1/S-arrest”, yet these cells exhibit some biochemical properties of S and G2 phase, such as cyclin E and cyclin B kinase activation and pRb hyperphosphorylation [13], [14], [15], [16]. One consequence of these events is the induction of E2F activator complexes following HCMV infection [17]. The RB-regulated activator class of proteins within the E2F family of transcription factors includes E2F1, E2F2, and E2F3a [18], [19]. These proteins regulate the transcription of many genes, such as those required for S-phase progression and DNA repair [19]. In addition, it has been shown that RB inactivation and deregulation of E2F1, but not E2F2 or E2F3, leads to DNA double strand break (DSB) accumulation and cell cycle checkpoint signaling [20], [21], [22], [23]. Although it is well established that one of the initial effects of HCMV infection is to inactivate the RB family of proteins, whether the consequential deregulation of the E2F proteins affects HCMV replication is unknown.

In this study, we asked whether there is a functional role for the host DDR in HCMV replication. We find that efficient HCMV replication requires a host DDR that centers on the presence of ATM and E2F1 protein. E2F2 and E2F3 do not influence the infection-associated DDR or viral replication. We show that expression of the HCMV IE proteins is sufficient to activate the host DDR. Our data suggests a model wherein HCMV infection stimulates an E2F1-mediated DDR to activate downstream pathways that facilitate the replication or maturation of nascent virus.

## Results

### HCMV Replication Is Compromised in Cells with Reduced Levels or Mutated ATM

**Figure 1:**
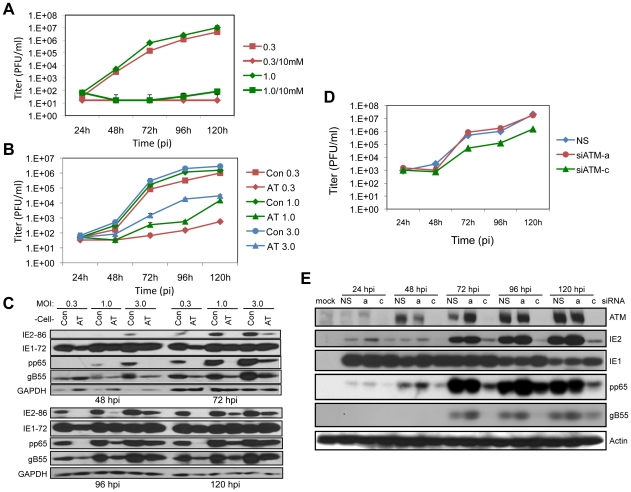
ATM is required for HCMV replication. (A) Caffeine blocks HCMV replication. HEL fibroblasts were infected at MOIs of 0.3 or 1.0 as noted in the figure. Cells were treated with 10 mM caffeine following virus absorption and the drug was replenished every 24 h. Cell supernatants were assayed for infectious virus production by plaque assay. The mean values are shown with bars denoting standard error for three independent experiments. (B) ATM is required for HCMV infection. Normal (Con) and AT dermal fibroblasts were infected at an MOI of 0.3, 1.0 or 3.0. Cell supernatants were assayed for infectious virus production by plaque assay. The mean values are shown with bars denoting standard error for three independent experiments. (C) Viral protein expression is altered in fibroblasts lacking ATM. Immunoblot analyses for IE (IE1/IE2), E (pp65) and L (gB55) HCMV protein expression in normal (Con) and AT dermal fibroblasts. (D) ATM depletion compromises HCMV replication. HEL fibroblasts were transfected with siRNAs specific for ATM (siATMa or siATMc) or with a control siRNA (NS) 24 h prior to infection with HCMV at an MOI of 0.1. Cell supernatants were assayed for infectious virus production by plaque assay. Note that siATMa did not deplete ATM. The mean values are shown with bars denoting standard error for three independent experiments. (E) Transient depletion of ATM alters viral expression. HEL fibroblasts were transfected with the indicated siRNA, and infected with HCMV at an MOI of 0.1. The levels of ATM and viral IE, E and L protein expression were assessed by immunoblot analysis for ATM, IE1/IE2, pp65 and gB55, respectively.

Many viruses require ATM activation for a fully permissive infection, and it has been reported that ATM is activated by IE1 expression or HCMV infection [8], [9], [10]. We asked whether HCMV replication is affected by functional changes in ATM. Initially, we examined the effects of caffeine, an inhibitor of PI3-like kinases including ATM, on HCMV replication. Following virus absorption, infected HEL fibroblasts were treated with 10 mM caffeine and virus yield was examined by plaque assay. As shown in Figure 1A, little or no virus replication was observed in the caffeine treated cells at either a low or moderate MOI, whereas HCMV replicated to expected levels in sham treated HEL fibroblasts. These results suggest that PI3-like kinase activity is necessary for HCMV replication.

Next we determined the contribution of ATM to HCMV replication by assessing viral replication in dermal fibroblasts from a normal donor compared to fibroblasts from a patient with ataxia telangiectasia (AT) that do not express detectable levels of the ATM protein. As shown in Figure 1B, much lower yields of infectious virus were generated in the AT fibroblasts compared to the control fibroblasts. The difference in HCMV replication was dose dependent with higher infectious doses (MOIs of 1.0 or 3.0) resulting in 2 to 3 log reductions in infectious virus production. Low MOI infection (MOI of 0.3) of AT fibroblasts resulted in little detectable viral progeny. At all time points and MOIs tested, infection of the AT fibroblasts resulted in reduced levels of IE2, pp65 and gB55, representing IE, E, and L viral gene products, respectively (Figure 1C). However, IE1 levels were not dramatically affected by the absence of ATM. Given that *UL123*, which encodes IE1, is the first viral gene expressed in infected cells, the lack of sustained changes in IE1 accumulation raises the possibility that later gene expression events are compromised. These results suggest that functional ATM is necessary for efficient HCMV replication.

Our observations suggesting a role for ATM in HCMV replication is contrary to another study [9]. Moreover, there is a concern with using AT fibroblasts as a model because the prolonged absence of functional ATM in cells from AT patients may have resulted in secondary genetic and/or biochemical changes that alter cellular environments and thereby influence HCMV replication. We addressed these issues by using siRNAs to transiently deplete ATM protein levels (siATM) in HEL fibroblasts. Cells were transfected with siATM 24 h prior to HCMV infection and viral replication (Figure 1D) and gene expression (Figure 1E) were monitored during a 5-day time course. Of the siRNAs designed to deplete ATM levels, only siATM-c was effective. This ATM-specific siRNA inhibited progeny virus production ∼10-fold throughout the replication time course (Figure 1D). Another siRNA, siATM-a, which did not consistently affect ATM levels, produced replication results comparable to a nonspecific siRNA (siNS). Similar to what we observed in dermal fibroblasts (Figure 1C), we found reduced levels of IE2, pp65 and gB55, but little change in IE1 levels when ATM levels were depleted by siATM-c (Figure 1E). We conclude that ATM is required for efficient HCMV replication.

### ATM Affects the Formation of Mature Replication Compartments

**Figure 2:**
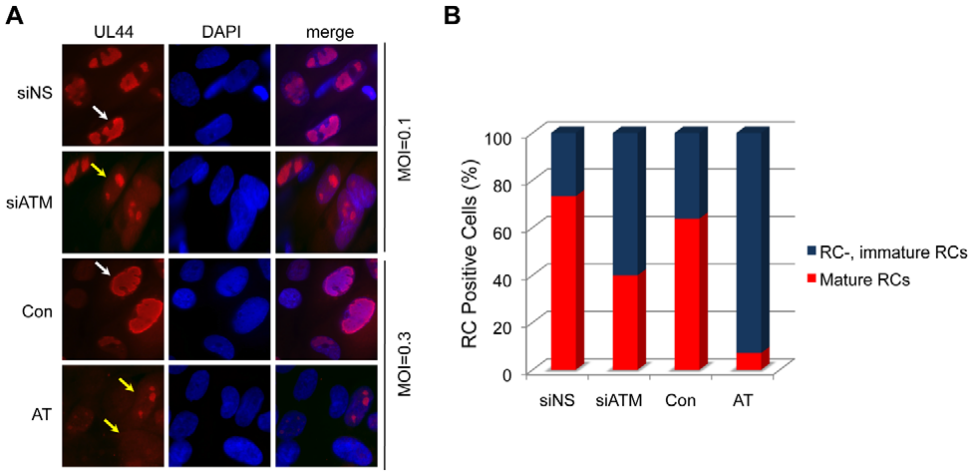
Reduced formation of “mature” viral replication compartments (RCs) in AT fibroblasts and siATM-transfected HEL fibroblasts. Normal (Con) and AT dermal fibroblasts were infected at an MOI of 0.3. HEL fibroblasts were transfected with control (NS) or siATMc, and subsequently infected with HCMV at an MOI of 0.1. Cells were fixed at 72 hpi and pUL44 detected by immunostaining. (A) Localization pUL44. Immunofluorescent images of normal (Con) and AT dermal fibroblasts infected with HCMV or HEL fibroblasts transfected with control (NS) or siATMc, subsequently infected with HCMV. Cells with “immature” RCs were defined as those with multiple, small pUL44 compartments (yellow arrows) and cells with “mature” RCs were identified as those composed of single, larger pUL44 compartments (white arrows). DAPI staining is used to define nuclei. (B) The percentage of fibroblasts with mature RCs was plotted relative to those lacking or having immature RCs. Over 200 cells were scored per sample.

We next determined whether cells deficient in ATM are compromised in the formation of replication compartments (RCs), which are sites of viral DNA replication and maturation. HEL fibroblasts were treated with siATM-c or control siRNA (siNS) and infected with HCMV and immunostained with anti-pUL44 antibody to detect HCMV replication compartments and scored (Figure 2). pUL44 is a virally encoded PCNA-like processivity factor of the viral DNA polymerase [24], [25]. In addition, dermal fibroblasts from normal and AT individuals were infected with virus, RC structures identified and scored. Under conditions of ATM depletion, the percentage of merged, “mature” RCs was reduced relative to control cells (Figure 2B). This difference was more apparent in AT fibroblasts where very few mature RCs were observed. The change in the percentages of mature RCs between siATM treated HEL fibroblasts and AT fibroblasts may explain why the replication phenotype observed in AT fibroblasts is dramatically different (compare viral replication curves in Figures 1B and 1D) whereas viral protein expression is less divergent (compare Figures 1C and 1E).

### ATM is Required for Host DNA Damage Signaling Early in Infection

**Figure 3:**
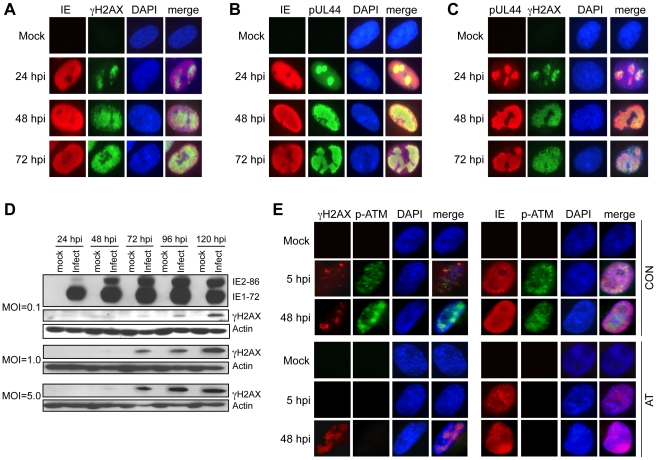
Accumulation and localization of host damage response proteins varies during infection. (A) γH2AX localization following HCMV infection. Immunofluorescent images of mock and virus-infected HEL fibroblasts (MOI = 1.0) are shown for IE and γH2AX localization. (B) Localization of HCMV IE and pUL44. Immunofluorescent images of mock and virus-infected HEL fibroblasts (MOI = 1.0) are shown for IE and pUL44 localization. (C) γH2AX localizes to HCMV replication compartments. Immunofluorescent detection of pUL44 and γH2AX in HCMV (MOI = 1.0) and mock-infected HEL fibroblasts is shown. (D) γH2AX accumulates during infection. HEL fibroblasts were infected with HCMV at the indicated MOI. Immunoblot analyses detected γH2AX protein in virus-infected fibroblasts. (E) ATM is required for γH2AX accumulation prior to the formation of viral DNA replication compartments. Immunofluorescent detection of γH2AX and phosphoserine 1981 ATM (p-ATM) or HCMV IE and p-ATM are shown in HCMV-infected cells fixed at 5 hpi and 48 hpi. Normal (Con) or AT dermal fibroblasts were infected with HCMV at an MOI of 5 and fixed at the indicated times pi. (A–C, E) DAPI staining is shown to identify nuclei.

Because it was reported that HCMV infection or expression of IE1 or IE2 can activate ATM as measured by autophosphorylation on Ser1981 [8], [10], we asked whether HCMV could induce the formation of DNA damage sensing foci containing γH2AX, an event downstream of ATM activation and other DNA damage-activated kinases [26], [27], [28]. γH2AX is the phosphorylated form of H2AX that is mediated by PI3-like kinases, including ATM. Infected cell cultures were co-immunostained for IE expression (both IE1 and IE2) to mark infected cells and γH2AX. As shown in Figure 3A, γH2AX staining was visible in the nuclei of cells expressing IE antigens. The levels of γH2AX protein increased over time and accumulated with infectious dose as measured by immunoblotting (Figure 3D).

The pattern of γH2AX immunostaining in Figure 3 is different from the punctate foci observed when cells are treated with DNA damaging agents that cause dsDNA breaks [28], [29]. In HCMV-infected cells, γH2AX appears to accumulate in larger “domains” of the nucleus and by 72 hpi, much of the nucleus appears to be reactive to the γH2AX antibody. Although this pattern of γH2AX immunostaining is unusual, it is reminiscent of viral RCs. To determine whether the γH2AX localization observed in infected cells is coincident with viral RCs, we co-immunostained infected cells for both γH2AX and pUL44. Although the IE proteins were not restricted to the RCs, γH2AX accumulated predominantly in these nuclear compartments based on co-immunostaining for pUL44 (Figures 3B–C). Thus, γH2AX accumulates in HCMV RCs.

One would anticipate that activated, autophosphorylated ATM would colocalize with γH2AX in RCs if ATM were responsible for γH2AX phosphorylation. Co-immunostaining for phosphoserine 1918-ATM and γH2AX in infected HEL fibroblasts showed that phosphoserine 1918-ATM and γH2AX colocalized at 24 hpi, but this pattern was diminished at 48–72 hpi (Figure S1). Next, we determined whether ATM is responsible for γH2AX accumulation following infection. As observed in HEL fibroblasts, infections of normal dermal fibroblasts showed partially overlapping co-immunostaining for phospho-ATM and γH2AX (Figure 3E). However, γH2AX still accumulated in AT dermal fibroblasts at 48 hpi in a pattern suggestive of co-localization in RCs. We also noticed that γH2AX accumulated in punctate foci early during infection (5 hpi) in both HEL and normal dermal fibroblasts (Figure S1 and Figure 3E). In contrast to the results observed at later times pi, no γH2AX was detected in AT dermal fibroblasts at 5 hpi (Figure 3E). Similar results were obtained in HEL fibroblasts when ATM was depleted with an siRNA (Figure S2). Therefore, ATM is responsible for the infection-associated DDR prior to the formation of mature RCs. However, this conclusion does not preclude the possibility that ATM may functionally contribute viral replication during other stages of the replication cycle.

### H2AX Contributes to HCMV Replication

**Figure 4:**
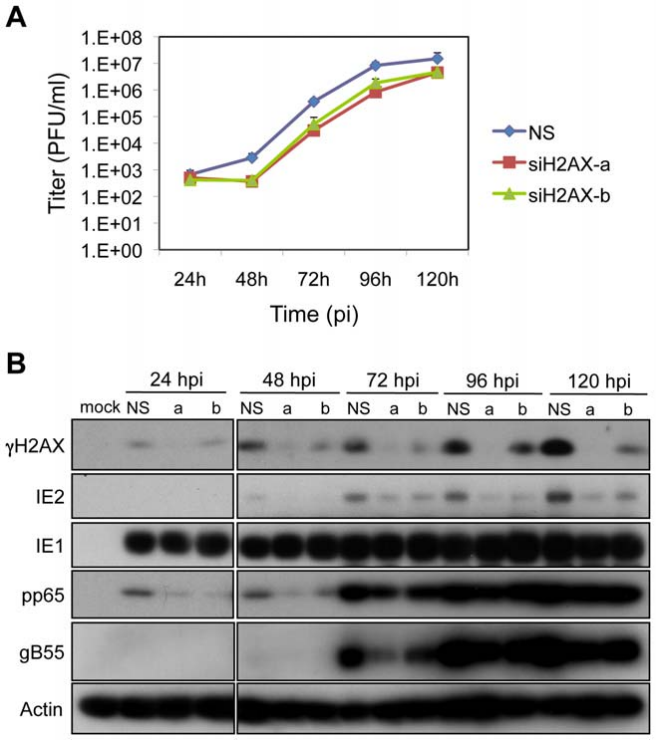
H2AX contributes to HCMV replication. (A) H2AX depletion compromises HCMV replication. HEL fibroblasts were transfected with siRNAs specific for H2AX (siH2AXa or siH2AXb) or with a control siRNA (NS) 24h prior to infection with HCMV at an MOI of 0.1. Cell supernatants were assayed for infectious virus production by plaque assay. (B) Depletion of H2AX alters viral protein accumulation. HEL fibroblasts were transfected with the indicated siRNA, and infected with HCMV at an MOI of 0.1. The levels of γH2AX and viral IE, E and L protein expression were assessed by immunoblot analysis for γH2AX, IE1/IE2, pp65, and gB55, respectively.

Given the contribution of ATM to viral replication, we determined whether downstream targets in the ATM-mediated DDR also influences replication. Here we focused on H2AX because it is responsive to ATM signaling (Figure 3E, and S2). Depletion of H2AX in HEL fibroblasts with either of two siRNAs reduced HCMV replication approximately 10 fold (Figure 4A). H2AX depletion also decreased the levels of IE2, and to a lesser extent pp65 and gB55 protein levels (Figure 4B). Another downstream target of ATM is p53. We had previously shown that p53 is phosphorylated by ATM during HCMV infection [8] and others have shown that p53 contributes to HCMV replication [12].

Phosphorylated H2AX may stabilize DNA damage recognition structures including MRE11-NBS1-RAD50 (“MRN”). We determined whether depletion of H2AX would impact the localization of NBS1 and DNA PK_CS_, a PI3-like kinase, during infection. HCMV infection appeared to increase the levels of both DNA PK_CS_ and NBS1 and the nuclear distribution of DNA PK_CS_ (Figure S3). The localization of NBS1 (Figure S3A) did not appear to be grossly impacted by reduced levels of γH2AX in infected cells. In contrast, the level and distribution of DNA PKcs (Figure S3B) appear to be similar to mock infected cells following treatment with siH2AX and HCMV infection (Figure S3). However, it is not apparent whether these patterns of protein localization are relevant to HCMV infection. While, H2AX, a cellular target of ATM-mediated signaling contributes to HCMV replication, the mechanism(s) by which this factor modulates replication is unclear.

### HCMV IE Protein Expression Induces the Nuclear Accumulation of γH2AX

**Figure 5:**
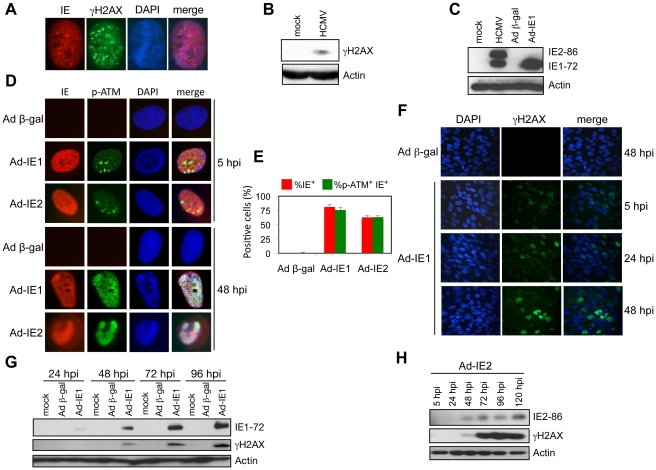
IE1 and IE2 expression induces a host DDR. (A) γH2AX foci form within 5 h of HCMV infection in HEL fibroblasts (MOI = 5). Immunofluorescent detection of HCMV IE and γH2AX proteins is shown. DAPI staining identifies the cell nuclei. (B) Accumulation of γH2AX protein at 5 h following HCMV infection. Immunoblot detection of γH2AX protein at 5 hpi (MOI = 5) is shown. (C) IE1-72 protein accumulation following transduction with Ad-IE1 into HEL fibroblasts. Immunoblot analysis of major HCMV IE proteins from whole cell lysates of HEL fibroblasts infected with HCMV (MOI = 0.1, 120 hpi) or transduced with either Ad-IE1 or Ad β-gal (MOI = 250, 48 hpi). HCMV IE proteins were identified with a monoclonal antibody that detects both IE1-72 and IE2-86 proteins. (D) Accumulation of p-ATM in HEL fibroblasts transduced with Ad-IE1 or Ad-IE2. Immunofluorescent detection of HCMV IE and p-ATM proteins is shown. DAPI staining identifies the cell nuclei. (E) Quantitation of the cells positive for IE or IE *plus* p-ATM observed in (D). Histograms show the average of three independent experiments and the error bars denote the standard deviation. (F) IE1 expression leads to nuclear γH2AX accumulation. HEL fibroblasts were transduced with Ad-IE1 or Ad β-gal and fixed for immunofluorescent detection of γH2AX at the indicated times post transduction. DAPI staining identifies nuclei. (G) Accumulation of γH2AX protein following IE1-72 expression. Immunoblot analysis for γH2AX protein in lysates of HEL fibroblasts transduced with Ad-IE1 or Ad β-gal and harvested at the indicated times post transduction. (H) Accumulation of γH2AX protein following IE2-86 expression. Immunoblot analysis for γH2AX protein in lysates of HEL fibroblasts transduced with Ad-IE2 and harvested at the indicated times post transduction.

Given the rapid formation of γH2AX foci and protein accumulation after HCMV infection (5 hpi; Figure 5A–B) and given that it has been previously reported that ectopic IE1 expression results in ATM autophosphorylation [8], we further examined the DDR associated with expression of IE gene products by monitoring the accumulation of γH2AX and p-ATM. Transduction of cells with Ad-IE1 or Ad-IE2 resulted in a time dependent nuclear accumulation of p-ATM and γH2AX (Figure 5C–H). Initially the p-ATM immunostaining pattern was punctate in the presence of IE1 or IE2 expression (Figure 5D). At 48 hpi, cells transduced with Ad-IE1 produced a broad, punctate pattern of p-ATM immunostaining, whereas p-ATM appeared to co-localize with IE2 in Ad-IE2 transduced cells (Figure 5D). Thus, ATM and H2AX phosphorylation occur early during HCMV infection and both IE1 and IE2 have the capacity to promote these events.

### E2F1 Contributes to Infection-Mediated DNA Damage Signaling

**Figure 6:**
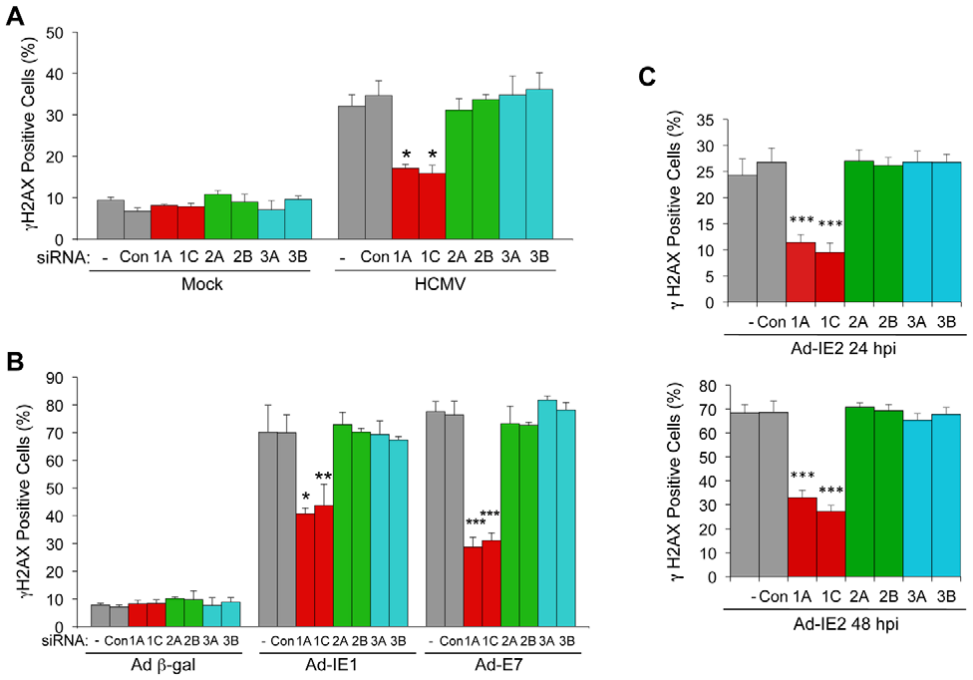
E2F1-specific contributes to the DDR associated with HCMV infection, IE1 or IE2 expression. (A) E2F1 contributes to the DDR following HCMV infection. Quantitation of γH2AX-positive cells in mock and HCMV-infected cells. HEL fibroblasts were transfected with siRNAs specific for E2F1 (1A, 1C), E2F2 (2A, 2B), or E2F3 (3A, 3B) or with a control siRNA (Con) 24 h prior to infection with HCMV at an MOI of 1.0. At 24 hpi, cells were fixed and γH2AX was detected by immunofluorescent staining. *P<0.003. (B) E2F1 contributes to the DDR induced by IE1. Quantitation of γH2AX-positive cells following transduction with recombinant adenoviruses. HEL fibroblasts were transfected with siRNAs as described in (A) 24 h prior to infection with a recombinant adenovirus that encodes Ad-IE1. At 24 hpi, cells were fixed and γH2AX detected by immunofluorescent staining. Transduction of cells with a recombinant adenovirus that encodes HPV-16 E7 (Ad-E7, MOI = 250) was used as a positive control for activation of the host DDR. *P<0.007; **P<0.02; ***P<0.001. (C) E2F1 contributes to the DDR induced by IE2. Quantitation of γH2AX-positive cells following siRNA transfection and transduction with Ad-IE2. At 24 hpi or 48 hpi, HEL fibroblasts were fixed and γH2AX detected by immunofluorescent staining. ***P<0.001. (A–C) Cells containing >2 γH2AX foci were scored as positive and plotted. Histograms indicate the average of three independent experiments and the error bars denote the standard deviation. *P* values were determined by Student's *t*-test.

We previously reported that altering RB function or increasing E2F1 levels leads to an ATM-dependent DDR [20], [21], [22], [30]. Given that HCMV infection or ectopic expression of IE1 or IE2 leads to increased E2F activity [17], [31], [32], [33], we asked whether E2F1 or other RB-associated, activator E2Fs were responsible for the DDR following HCMV infection or IE cDNA transduction. To address this question, we individually blocked the expression of *E2F1, E2F2* or *E2F3* with one of two different siRNAs prior to infection with HCMV or transduction with recombinant adenoviruses, and then scored cells for a host DDR by γH2AX immunostaining. A low basal percentage (<10%) of HEL fibroblasts stained positive for γH2AX in mock-infected HEL cells (Figure 6A). This level of γH2AX immunostaining most likely represents DNA damage signaling that normally occurs in human fibroblasts replicating their own DNA [30]. Depletion of individual E2Fs did not affect this background staining (Figure 6A). Infection with HCMV resulted in increased γH2AX immunostaining, with ∼35% of the cells positive for γH2AX (Figure 6A). This percentage dropped to ∼16% when either of two siRNAs targeting *E2F1* expression was transfected into cells prior to infection. Depletion of either E2F2 or E2F3 with specific siRNAs did not significantly alter the percentage of cells staining positive for γH2AX. Transduction with a control recombinant adenovirus encoding β-gal did not affect the background of levels of γH2AX staining, but transduction with Ad-IE1 resulted in the majority of HEL fibroblasts immunostaining positive for γH2AX (Figure 6B). Only depletion of E2F1 reduced the percentage of γH2AX-positive cells, depleting E2F2 or E2F3 with siRNAs had no effect on the host DDR (Figure 6B). Likewise, only E2F1 depletion reduced the percentage of γH2AX-positive HEL fibroblasts when transduced with AD-IE2 (Figure 6C). Multiple time points are shown for this experiment because of the lower percentages of γH2AX-positive cells at earlier times post transduction. Ad-E7, which encodes HPV type 16 E7, was included as a positive control for E2F1-mediated DDR [20], [21]. Therefore, HCMV infection and IE1 or IE2 expression activate an E2F1-mediated DNA damage response.

### E2F1 Contributes to HCMV Replication

**Figure 7:**
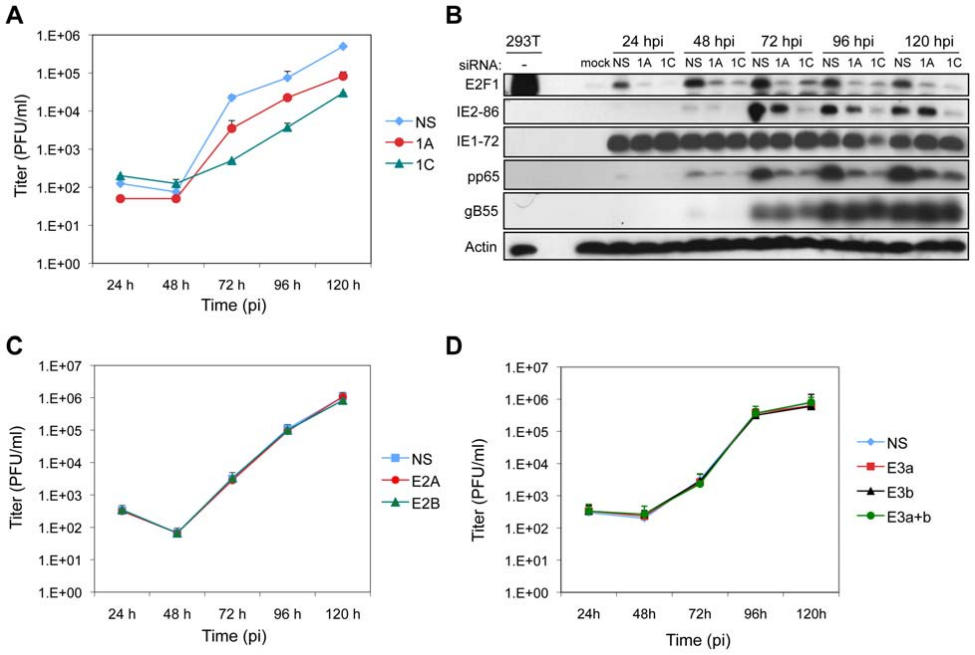
E2F1 is required for efficient virus replication. (A) Production of progeny virus in HEL fibroblasts following transfection with siRNAs that deplete E2F1 levels (1A or 1C) or with a control siRNA (NS) 24 h prior to HCMV infection (MOI = 0.1). Culture supernatants from infected cells were assayed for infectious virus production by plaque assay. (B) Expression of E2F1 and HCMV proteins during infection. E2F1 and markers of viral IE, E, and L protein expression were detected by immunoblotting of cells lysates from (A). (C) Production of progeny virus in HEL fibroblasts following transfection with siRNAs that deplete E2F2 levels (E2A or E2B) or with a control siRNA (NS) 24 h prior to HCMV infection (MOI = 0.1). Samples processed as described in (A). (D) Production of progeny virus in HEL fibroblasts following transfection with siRNAs that deplete the levels of E2F3a (E3a), E2F3b (E3b), or both E2F3a and E2F3b (E3a+b), or with a control siRNA (NS) 24 h prior to HCMV infection (MOI = 0.1). Samples processed as described in (A).

Given the observations that ATM is required for HCMV replication and that E2F1 contributes to the DDR in infected and IE1 or IE2 transduced cells, we next determined whether E2F1 specifically contributes to HCMV replication. HEL fibroblasts were transfected with siRNAs specific for E2F1 or with a control siRNA 24 h prior to infection with HCMV. Although virus infection increases the levels of E2F1, depletion of E2F1 in infected cells reduced its levels approaching that observed in the mock-infected sample (Figure 7B). Transfection of either E2F1-specific siRNA also reduced viral IE, E and L gene expression as measured by immunoblotting for IE2, pp65 and gB55, respectively. However, IE1 levels were not consistently affected by E2F1 depletion. Depletion of E2F1 also altered HCMV replication with a ∼5 to ∼50-fold reduction in progeny virus production that was dependent on the siRNA used to deplete E2F1 levels (Figure 7A). These results are consistent with the patterns observed for ATM depletion (Figures 1D–E).

E2Fs are generally thought to function as transcription factors with E2F1 having additional, less well-characterized roles in DNA damage accumulation and apoptosis [34]. To begin to differentiate whether the effects of E2F1 depletion on virus replication were due to reduced levels of an “activator E2F” (i.e., E2F1, E2F2, and E2F3a) or due to unique functions of E2F1, we determined whether depletion of E2F2 or E2F3 would affect HCMV protein expression and replication. Depletion of E2F2, E2F3a, E2F3b (an E2F3 isoform that does not contribute to proliferation [35]), or a combination of E2F3a and E2F3b reduced the levels of the targeted protein to approximately that observed in mock-infected samples (Figures S4A–B). Targeting of E2F2 or E2F3a or E2F3b, or the combination of E2F3a and E2F3b had no discernable effect on the accumulation of viral proteins (Figure S4A–B) or the production of progeny virus (Figures 7C–D). These results suggest that the specific deregulation of E2F1 levels is required for efficient replication of HCMV.

## Discussion

**Figure 8:**
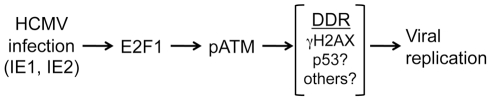
Model showing the relationship between infection, E2F1, ATM activation and HCMV replication.

In this study, we find that HCMV infection stimulates an E2F1-mediated DDR that centers on activation of the ATM kinase early in infection and subsequently coordinates with nuclear viral replication compartments. Moreover, we show that ATM and downstream signaling are required for replication following infection at a low MOI and contributes to HCMV replication at higher doses (Figure 8). Our results are consistent with ATM contributing to the replication of other viruses (for review, see [36]). This conclusion contrasts with what has been previously reported for HCMV infection [9], where it was determined that ATM is not required for the progression of HCMV infection. It is unclear why there is a discrepancy between these studies, but we have confirmed our results using multiple approaches (Figure 1).

It appears that ATM activation represents a general response to infection by DNA viruses or viruses that have a DNA stage in the replication strategy, such as retroviruses. The question remains as to why viruses activate ATM and other DDR proteins for replication. Indeed, activation of the host DDR is an obstacle for the replication of at least one DNA virus, adenovirus, which blocks the host DDR during infection [37]. One reason for infection-associated ATM activation may be to utilize the consequential stimulation of cellular DNA repair and recombination enzymes [38] to benefit viral replication [3], [39], [40]. Perhaps, in the case of HCMV, repair and recombination enzymes may aid in circularizing the viral DNA after it has entered the cell and/or facilitate the maturation of nascent viral genomes. A DNA repair complex of DNA ligase IV and XRCC4 circularizes herpes simplex virus genomes early in infection [41]. If correct, one would predict that γH2AX, as well as other DDR factors would be bound to virion-delivered HCMV DNA once uncoated in the nucleus. However, others have concluded that parental viral DNA and γH2AX do not co-localize [9]. It remains an open question as to whether there is a contribution of the host DDR to very early events in HCMV replication.

HCMV gene expression patterns in infected cells lacking or depleted for ATM may offer clues to the stage(s) in infection that depend on ATM function. IE1 expression is largely unaffected by ATM status. One interpretation of this observation is that ATM does not influence IE events that affect viral replication. Our observation that mature RCs and marker E and L gene products are reduced during infection of AT fibroblasts is consistent with a model wherein ATM influences events associated with DNA replication, presumably by stimulating host (or viral) factors to aid in the repair or recombination of nascent viral DNA. A role for ATM in DNA repair or recombination post replication is also a possibility, although the pattern of viral gene expression argues against this idea. It also cannot be excluded that ATM may have a novel function in phosphorylating/activating an essential host or viral factor not associated with DNA replication, repair, or recombination.

A number of mechanisms have been proposed for how viral infections lead to ATM activation. Upon HIV infection, ATM activation requires the viral integrase and it is proposed that ATM functions in post integration DNA repair [41]. For polyomaviruses like SV40, it is thought that the onset of viral DNA replication activates ATM, which then phosphorylates an essential serine residue on large T antigen [42]. HPV genome replication appears to switch from theta to rolling circle replication [43], which may activate ATM. Alternately, infection by DNA viruses may cause damage to host chromosomes, which would stimulate a host DDR. In this situation, targets of ATM phosphorylation should also contribute to viral replication.

Both H2AX (Figure 3) and p53 [8] are substrates of the ATM kinase during HCMV infection and both H2AX (Figure 4) and p53 [12] contribute to HCMV replication. While the mechanism by which H2AX contributes to HCMV replication is unknown, p53 is found in RCs, binds viral DNA and evidence suggests that p53 influences the expression of viral genes [44]. However, the roles of ATM-mediated phosphorylation of H2AX or p53 to productive replication are not known at this point.

Somewhat surprisingly, we find that the initial accumulation of γH2AX following HCMV infection is dependent on ATM whereas ATM is dispensable for γH2AX accumulation once mature viral DNA replication compartments are formed. The kinase(s) responsible for phosphorylating H2AX in the absence of ATM at these later times is unknown but it is possible that another PI3-like kinase, perhaps ATR [45], is responsible for H2AX phosphorylation. DNA PKcs, another PI3-like kinase, is known to phosphorylate H2AX in response to DNA damage signaling, but it has been shown that DNA PKcs does not localize to HCMV RCs [9]. However, even though H2AX can be phosphorylated by other kinases later during infection, activated ATM is mostly located in HCMV RCs at these times pi, leaving open the possibility that ATM is influencing activities in these nuclear compartments.

ATM is required for efficient H2AX phosphorylation in MHV68-infected primary marcrophages and ATM is relocalized to sites of viral genome deposition, although a viral kinase also contributes to H2AX phosphorylation [46]. ATM is also rapidly relocalized to replication compartments during HSV infection [3]. It will be interesting to determine whether ATM is only transiently responsible for host DDR signaling (and viral replication) during infections with herpesviruses.

Deregulation of E2F activity is a hallmark of infections with many DNA viruses that replicate in the nucleus. HCMV infection and expression of its major IE proteins, particular IE1 and IE2, have been shown to inactivate RB family members and induce the expression of E2F regulated genes [17], [33], [47], [48] possibly by providing host factors that contribute to virus replication. Our data reveal another consequence of inactivating RB family members and the specific deregulation of E2F1, the activation of a host DDR that facilitates the replication of HCMV.

The mechanism by which E2F1 stimulates host DDR is not known. Inactivation of RB and the subsequent deregulation of E2F1—but not the related family members, E2F2 or E2F3, which also interact with RB—leads to an accumulation of DNA double-strand breaks in human fibroblasts [21]. Although it is not clear if HCMV infection causes extensive host DNA damage, infection can result in a DNA double strand break on chromosome 1 [49]. Whether this single DNA break is sufficient to initiate the observed host DDR is unclear. Alternately, it has been shown that activation of a DDR does not necessarily require DNA lesions. Rather, the physical interaction of DNA repair factors with chromatin can be sufficient to activate the DDR signaling cascade [50]. Therefore, host chromosomal changes mediated by disruption of RB/E2F1 complexes or other mechanisms of E2F1 deregulation should also be considered as possible ATM activators during infection.

Most productive infections by DNA viruses result in deregulation of E2F activity through inactivation of RB and RB family members [51]. These viruses also activate an ATM-centric DDR, although some viruses, including MHV68, KSHV, and adenovirus, encode factors that can block signals from reaching ATM or its targets [37], [52], [53], [54]. The herpesviral proteins responsible for this inhibition are often expressed during latency, which raises the possibility that the host DDR interferes with aspects of latency such as cell survival, proliferation or, perhaps, the maintenance of viral episomes. Most of these viruses have in common infection-associated E2F deregulation, DDR activation, and a contribution of ATM to productive infections. These shared features raise the possibility that E2F1 contributes to the replication of many viruses through its activation of the ATM-associated DDR. It will be interesting to determine how common the E2F1-mediated DDR is to productive viral infections.

## Materials and Methods

### Cell Culture

AT dermal fibroblasts from an ataxia-telangiectasia patient (GM05823C; termed “AT”), age-matched primary human dermal fibroblasts (GM00316B; termed “CONB”) and human embryonic lung fibroblasts (HEL fibroblasts) were obtained from the Coriell Institute for Medical Research (Camden, N.J). Dermal fibroblasts were maintained in Minimum Essential Media (MEM) supplemented with 15% fetal bovine serum (FBS) and 1% penicillin-streptomycin. HEL fibroblasts were cultured in Dulbecco modified Eagle medium (DMEM) supplemented with 10% FBS and 1% penicillin-streptomycin. All media, FBS, and antibiotics were from GIBCO.

### HCMV and Infections

HCMV strain AD169 was obtained from the American Type Culture Collection (ATCC, Manassas, VA). Fibroblasts were infected with HCMV AD169 at various multiplicities of infection (MOI). Viral infections were performed in growth media with 2% FBS for 2 hours. The viral inoculum was removed and replaced with normal grow medium. Cells pellets were collected at different time post infection and lysates were generated as described as below.

### Drug Treatment

Cells were treated with caffeine (Sigma) at a dose of 10 mM following virus absorption. The drug was replenished every 24 h.

### Adenovirus Vectors and Infection

Recombinant adenoviruses encoding HCMV IE1-72 (Ad-IE1), HCMV IE2-86 (Ad-IE2), β-galactosidase (Ad β-gal), and HPV16 E7 (Ad-E7) have been described [11], [20], [55], [56]. Recombinant adenovirus stocks were generated, purified and titered as described [20], [57]. All recombinant adenovirus infections were done at a MOI of 250 unless otherwise noted.

### siRNA and Transfections

siRNA were transfected at 50–100 nM using Lipofectamine 2000 (Invitrogen) or by electroporation in the presence of siPort transfection buffer (Ambion). The nonspecific siRNAs (NS) were composed of a nonsense sequence and had no effect on parameters tested relative to mock transfection. Transfection conditions for individual siRNAs were optimized. The sequences of the siRNAs used in this study are as follows:

siNS (5′-CTTCCTCTCTTTCTCTCCCTTGTGA-3′) [used as a control for siATM],

siATM-a (5′-GGAGTTATTGATGACGTTACATGAG-3′),

siATM-c (5′-CGCATGTGATTAAAGCAACATTTGC-3′),

siE2F1A (5′-GGCCCGATCGATGTTTTCC-3′),

siE2F1C (5′-GTCACGCTATGAGACCTCA-3′),

siE2F2A (5′-GTGCATCAGAGTGGATGGC-3′),

siE2F2B (5′-CAAGAGGCTGGCCTATGTG-3′),

siE2F3a (5′-GCGTACATCCAGATCCTCA-3′),

siE2F3b (5′-GGAAATGCCCTTACAGCAG-3′),

siE2F3(a+b) (5′-GACCAAACTGTTATAGTTG-3′),

siH2AXa (5′-CAACAAGAAGACGCGAATC-3′)

siH2AXb (5′-CGACGAGGAGCTCAACAAG-3′)

NS (5′-TTTTTTTCCCCAAAGGGGG-3′) [used as a control for siE2F and siH2AX treatments].

### Viral Growth Curves

Cells were seeded and infected at the listed MOI for each experiment. Triplicate infections were performed for each time point. At the indicated times pi, a small aliquot (200 ul) of supernatant was harvested from each dish and stored at −80°C. Viral titers were then determined on HEL fibroblasts using standard techniques. Plaques were counted 7 dpi using Giemsa stain (Sigma) to enhance the visualization of plaques. Plotted values represent the average of triplicate infections.

### Immunoblot Analysis

Infected cells were harvested at the indicated time point and pellet cells were stored at −80°C. Thawed cell pellets were resuspended in radioimmunoprecipitation assay buffer (RIPA: PBS, 0.1%NP-40, 1% sodium dodecyl sulfate, 0.5% sodium deoxycholate, sodium vanadate, phenylmethylsulfonyl fluoride, and aprotinin) and incubated on ice for 1 h. Samples were sonicated for 15 sec, and soluble proteins were collected by centrifugation for 10 min at 13,000 rpm in a microcentrifuge. Proteins were resolved by SDS-PAGE, and the proteins were transferred to a polyvinylidene difluoride membrane (Perkin-Elmer) by electroblotting. Detection of E2F1, E2F2, E2F3, IE, pp65, gB55, ATM, γH2AX and actin proteins was performed with antibodies specific for E2F1 (C-20, Santa Cruz Biotechnology), E2F2 (C-20, Santa Cruz Biotechnology), E2F3 (C-18, Santa Cruz Biotechnology), IE1-72 and IE2-86 (MAB8130, Chemicon International), pp65 (CA003-100, Virusys), gB55 (Shan Lu, UMass Medical School), ATM (D2E2, Cell Signaling), histone γH2AX (Upstate Biotechnology), actin (A5316, Sigma) and HRP-conjugated secondary antibodies. Protein bands were visualized by chemiluminescence with ECL reagent (Amersham).

### Immunofluorecence Analysis

Cells were plated on glass coverslips that were pretreated with 40% HCl for 2 min followed by a 5 min wash in 70% ethanol. Cells were infected with recombinant adenoviruses or HCMV at the indicated MOIs. Cells were washed three times with PBS and fixed with 2% paraformaldehyde. Fixed cells were blocked in 10% FBS for 1 h at room temperature and incubated with antibodies against IE (MAB8130, Chemicon International), pUL44 (Virusys), γH2AX (Upstate Biotechnology), phospho-Ser1981 ATM (Rockland Immunochemicals), NBS1 (GeneTex), and DNA PKcs (Thermo Scientific). FITC conjugated goat anti-rabbit, Texas Red-conjugated goat anti-mouse IgG1 or IgG2a secondary antibodies (Southern Biotechnology Associates, Inc) were used to detect bound primary antibody by immunofluorescence. Images were captured on a Nikon microscope and analyzed using Improvision software. Over 200 cells were counted per sample when quantifying cell staining.

### Statistical Analysis

Statistical analyses were performed using unpaired *t*-tests. Values are expressed as mean ± SD of three independent experiments. A *P* value of ≤0.05 was considered statistically significant.

## Supporting Information

Figure S1 Localization of DDR proteins in HEL fibroblasts during HCMV infection. (A) Immunofluorescent detection for phospho-serine 1981ATM (p-ATM) and γH2AX in mock and virus-infected HEL fibroblasts (MOI = 1.0). (B) Immunofluorescent detection for phospho-serine 1981 ATM (p-ATM) and IE1/IE2 protein in mock and virus-infected HEL fibroblasts (MOI = 1.0). (A–B) DAPI staining identifies nuclei.
(2.35 MB PNG)

Figure S2 ATM is required for γH2AX accumulation early in infection. Immunofluorescent detection of γH2AX and phospho-serine 1981 ATM (p-ATM) or IE1-72 and p-ATM is shown in HCMV-infected cells fixed at 5 hpi and 48 hpi. HEL fibroblasts were transfected with siRNAs specific for ATM (siATMc) or with a control siRNA (NS) 24 h prior to infection with HCMV at an MOI of 5.0. DAPI staining identifies nuclei.
(1.79 MB TIF)

Figure S3 Depletion of H2AX does not affect the localization of NBS1 but alters the distribution of DNA PKcs in infected cells. HEL fibroblasts were transfected with control (NS) or H2AXa siRNA and infected with HCMV at an MOI of 1.0. Cells were fixed at 48 h post infection. The level of expression and localization of γH2AX, together with NBS1 (A) or DNA PKcs (B) in mock and virus-infected cells were detected by immunostaining. DAPI staining identifies nuclei.
(0.98 MB TIF)

Figure S4 Depletion of E2F2 or E2F3 does not affect viral protein expression patterns. (A) Expression of HCMV proteins during infection in the presence of siRNAs against E2F2 expression. HEL fibroblasts were transfected with siRNAs specific for E2F2 (E2A or E2B) or with a control siRNA (NS) 24 h prior to infection with HCMV (MOI = 0.1). E2F2 and markers of HCMV IE, E and L proteins were detected by immunoblotting. (B) Expression of HCMV proteins during infection in the presence of siRNAs against E2F3a, E2F3b expression or an siRNA that reduces the expression of both E2F3a and E2F3b. HEL fibroblasts were transfected with siRNAs specific for E2F3a (E3a), E2F3b (E3b), the combination of E2F3a and E2F3b (E3a+b), or with a control siRNA (NS) 24 h prior to infection with HCMV (MOI = 0.1). E2F3 and markers of HCMV IE, E and L proteins were detected by immunoblotting.
(1.12 MB TIF)
