# Supplementary figures and images for: An E2F1-Mediated DNA Damage Response Contributes to the Replication of Human Cytomegalovirus
Source: PLoS Pathog. 2011 May 12;7(5):e1001342. doi: 10.1371/journal.ppat.1001342 (PMC3093362; doi:10.1371/journal.ppat.1001342)

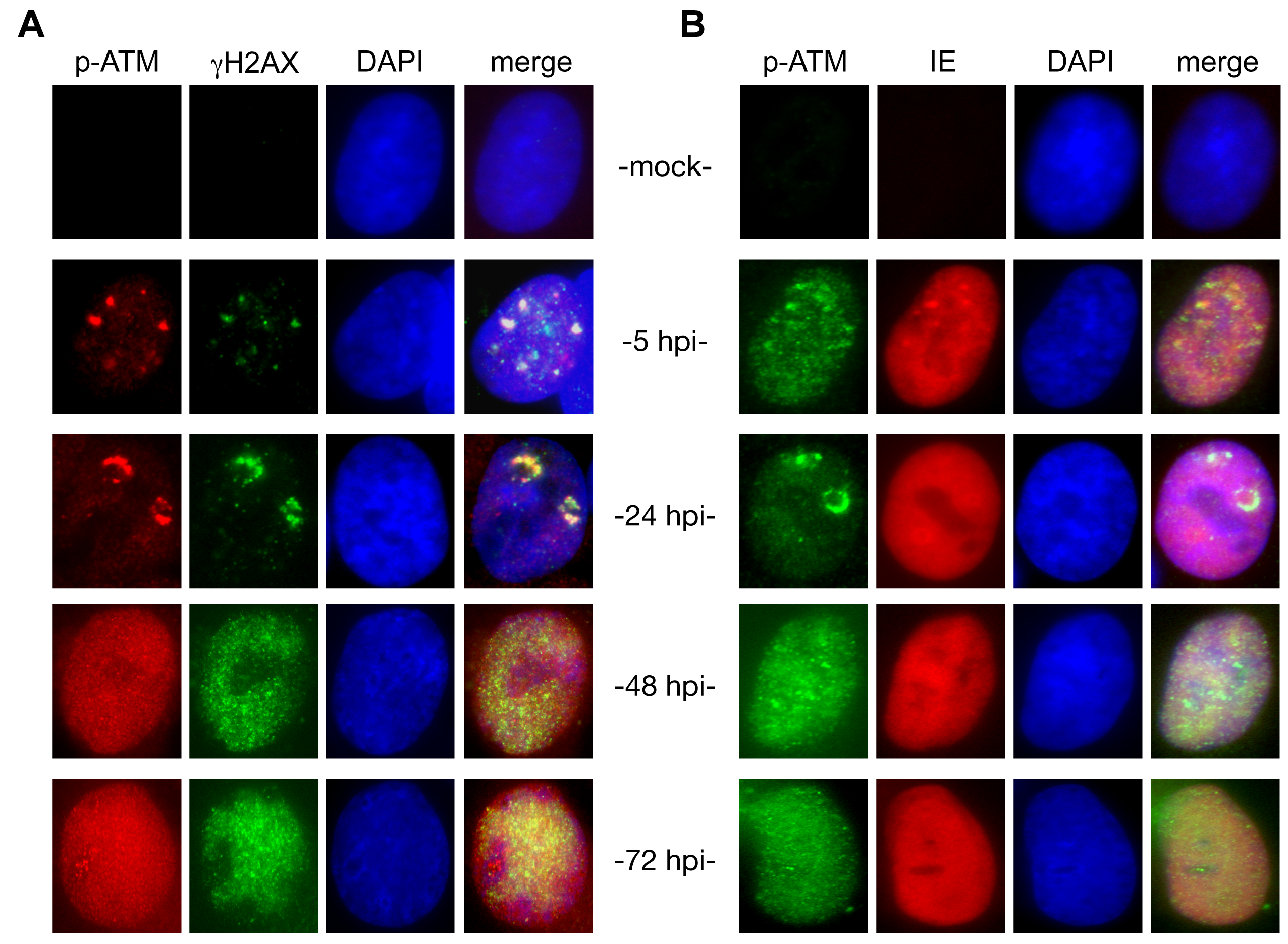

Supplement: Figure S1 — Localization of DDR proteins in HEL fibroblasts during HCMV infection. (A) Immunofluorescent detection for phospho-serine 1981ATM (p-ATM) and γH2AX in mock and virus-infected HEL fibroblasts (MOI = 1.0). (B) Immunofluorescent detection for phospho-serine 1981 ATM (p-ATM) and IE1/IE2 protein in mock and virus-infected HEL fibroblasts (MOI = 1.0). (A–B) DAPI staining identifies nuclei. (2.35 MB PNG) [file ppat.1001342.s001.png]

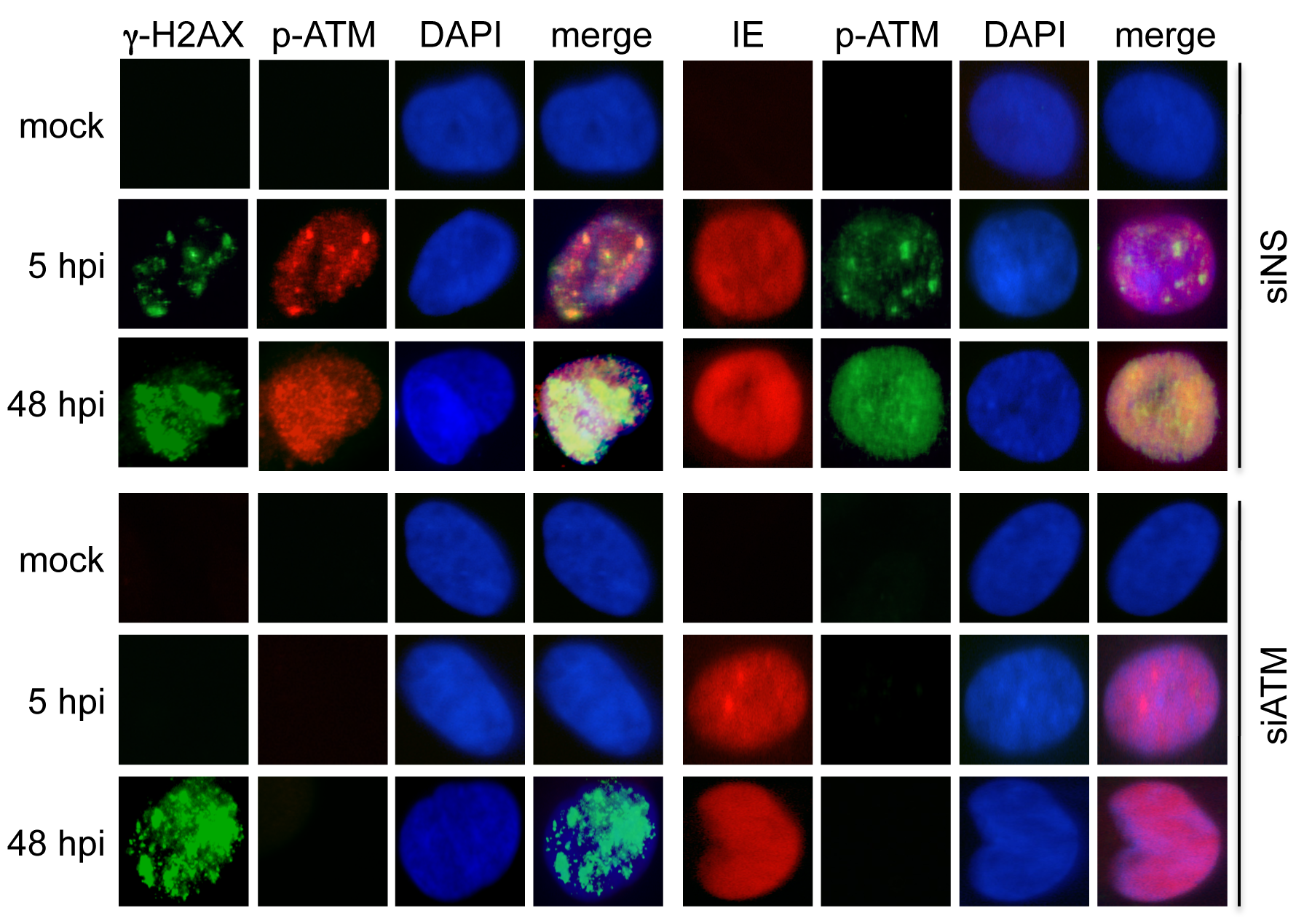

Supplement: Figure S2 — ATM is required for γH2AX accumulation early in infection. Immunofluorescent detection of γH2AX and phospho-serine 1981 ATM (p-ATM) or IE1-72 and p-ATM is shown in HCMV-infected cells fixed at 5 hpi and 48 hpi. HEL fibroblasts were transfected with siRNAs specific for ATM (siATMc) or with a control siRNA (NS) 24 h prior to infection with HCMV at an MOI of 5.0. DAPI staining identifies nuclei. (1.79 MB TIF) [file ppat.1001342.s002.tif]

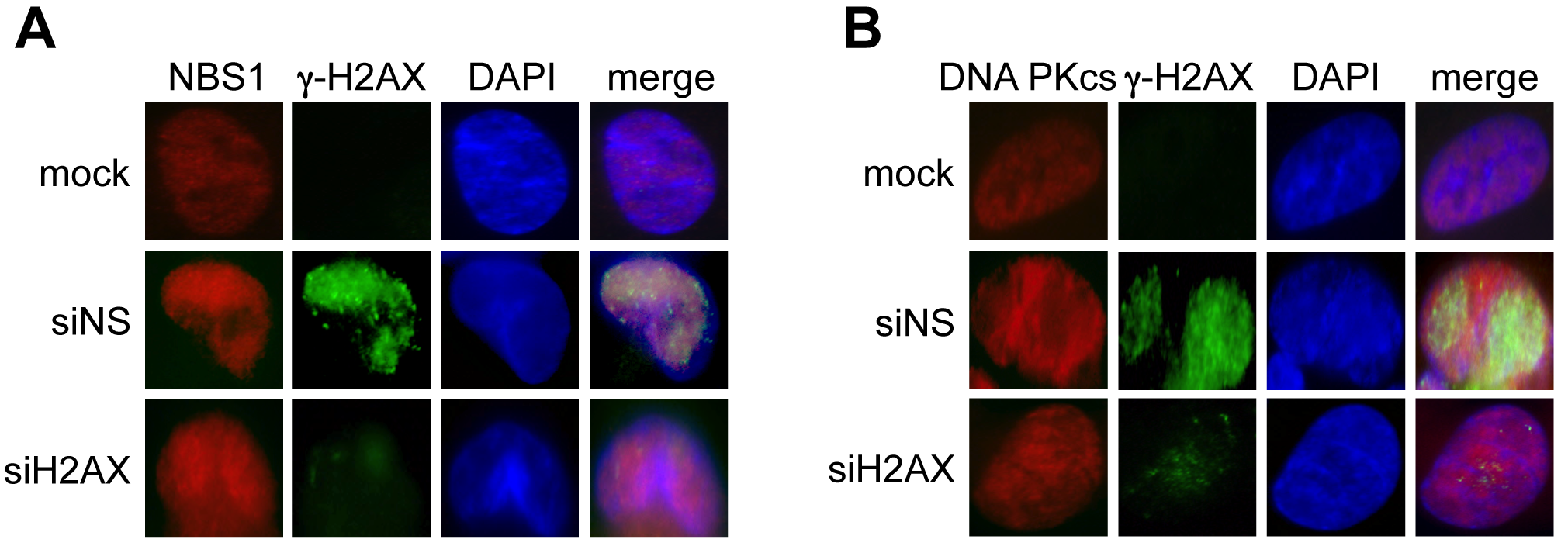

Supplement: Figure S3 — Depletion of H2AX does not affect the localization of NBS1 but alters the distribution of DNA PKcs in infected cells. HEL fibroblasts were transfected with control (NS) or H2AXa siRNA and infected with HCMV at an MOI of 1.0. Cells were fixed at 48 h post infection. The level of expression and localization of γH2AX, together with NBS1 (A) or DNA PKcs (B) in mock and virus-infected cells were detected by immunostaining. DAPI staining identifies nuclei. (0.98 MB TIF) [file ppat.1001342.s003.tif]

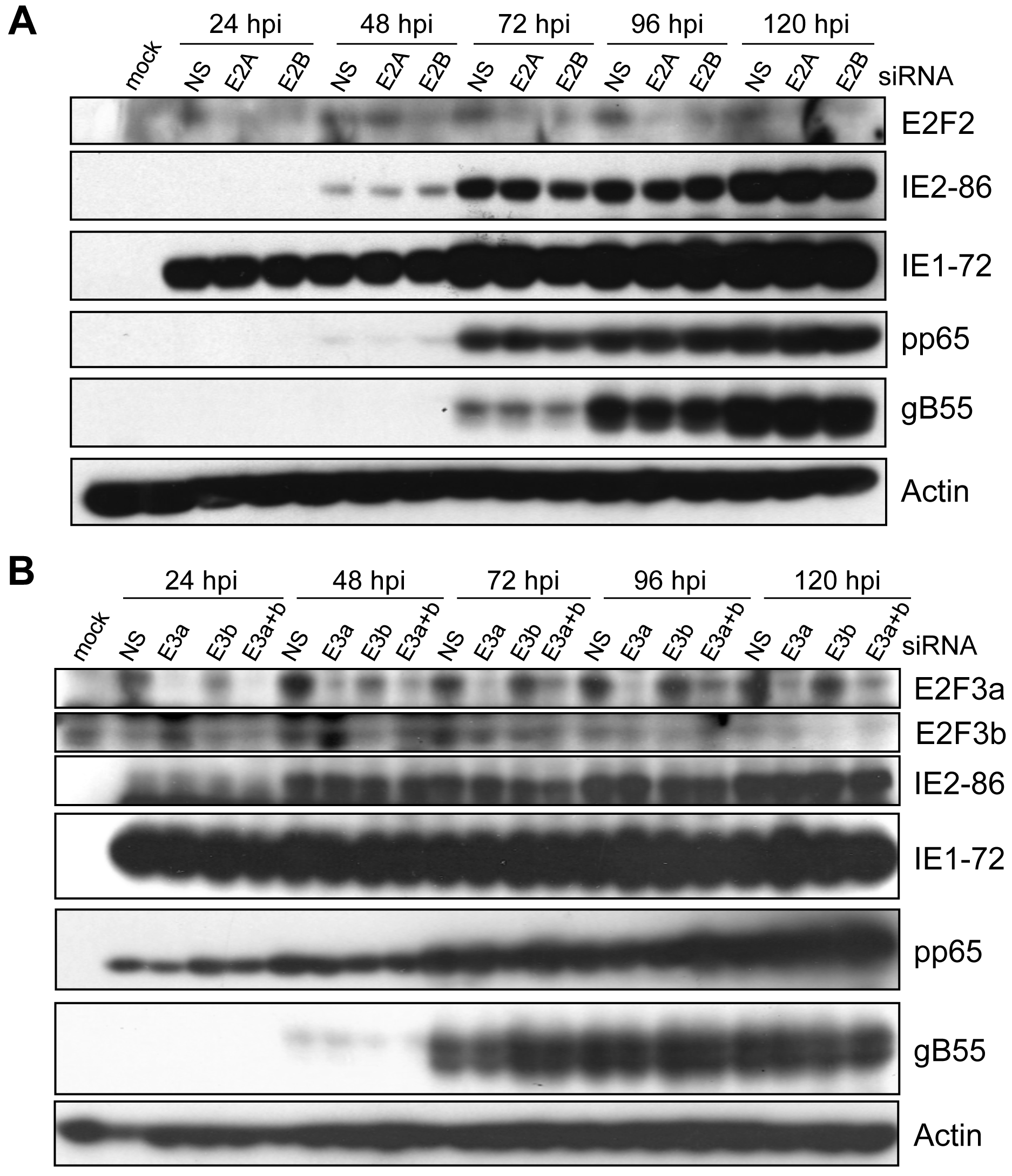

Supplement: Figure S4 — Depletion of E2F2 or E2F3 does not affect viral protein expression patterns. (A) Expression of HCMV proteins during infection in the presence of siRNAs against E2F2 expression. HEL fibroblasts were transfected with siRNAs specific for E2F2 (E2A or E2B) or with a control siRNA (NS) 24 h prior to infection with HCMV (MOI = 0.1). E2F2 and markers of HCMV IE, E and L proteins were detected by immunoblotting. (B) Expression of HCMV proteins during infection in the presence of siRNAs against E2F3a, E2F3b expression or an siRNA that reduces the expression of both E2F3a and E2F3b. HEL fibroblasts were transfected with siRNAs specific for E2F3a (E3a), E2F3b (E3b), the combination of E2F3a and E2F3b (E3a+b), or with a control siRNA (NS) 24 h prior to infection with HCMV (MOI = 0.1). E2F3 and markers of HCMV IE, E and L proteins were detected by immunoblotting. (1.12 MB TIF) [file ppat.1001342.s004.tif]
